# Managerial attitudes and perceived barriers regarding evidence-based practice: An international survey

**DOI:** 10.1371/journal.pone.0184594

**Published:** 2017-10-03

**Authors:** Eric Barends, Josh Villanueva, Denise M. Rousseau, Rob B. Briner, Denise M. Jepsen, Edward Houghton, Steven ten Have

**Affiliations:** 1 Center for Evidence Based Management (CEBMa), Amsterdam, The Netherlands; 2 Claremont Graduate University, Claremont, California, United States of America; 3 Heinz College, Carnegie Mellon University, Pittsburgh, Pennsylvania, United States of America; 4 School of Business and Management, Queen Mary University of London, London, United Kingdom; 5 Faculty of Business and Economics, Macquarie University, North Ryde, Australia; 6 Human Capital and Metrics, Chartered Institute of Personnel & Development (CIPD), London, United Kingdom; 7 VU University of Amsterdam, Amsterdam, The Netherlands; Universitat Zurich, SWITZERLAND

## Abstract

Evidence-based practice (EBP) in management is still in its infancy. Several studies suggest that managers in businesses and other organizations do not consult the scientific evidence when making decisions. To facilitate its uptake, we need to better understand practitioner attitudes and perceived barriers related to EBP. In medicine and nursing, an abundance of research exists on this subject, although such studies are rare in management. To address this gap, we surveyed 2,789 management practitioners in Belgium, the Netherlands, the United States, the United Kingdom and Australia. Our findings indicate that most managers we studied have positive attitudes towards EBP. However, lack of time and a limited understanding of scientific research are perceived as major barriers to the uptake and implementation of EBP in management. Studies in other professions where EBP is far more established also report similar barriers. We discuss the implications of our findings for practice, education and research, providing suggestions to enhance use of EBP in management practice.

## Introduction

Evidence-based practice means making decisions through the conscientious, explicit and judicious use of the best available evidence from multiple sources to increase the likelihood of a favorable outcome [1]. The term ‘evidence-based’ was coined in the 1990s in medicine, though its principles now extend across disciplines as varied as nursing, education, criminology, social work, and public policy. In management, however, evidence-based practice (EBP) remains in its infancy; A substantial amount of research suggests that managers do not read academic articles [2–6] or consult the scientific evidence [3, 7–9]. As a result, managers are often not aware of the accumulated scientific evidence available on key issues in their practice. For example, a survey of 950 American HR professionals showed large discrepancies between what managers think is effective and what the body of scientific research shows [5]. This study’s findings have been replicated in other countries [10, 11]. Ignorance regarding scientific findings relevant to management practice is comparable to that of medicine 25 years ago–Gordon Guyatt, who coined the term ‘evidence-based’ in 1990, noted: ‘The problem isn’t clinical experience: the problem is that we (physicians) are so unsystematic, intuitive, and with no notion of scientific principles in our accumulation of clinical experience’ [12]. Yet managerial decisions affect working lives and well-being around the world. As Henry Mintzberg said, ‘No job is more vital to our society than that of a manager. It is the manager who determines whether our social institutions serve us well or whether they squander our talents and resources.’ [13].

**Fig 1:**
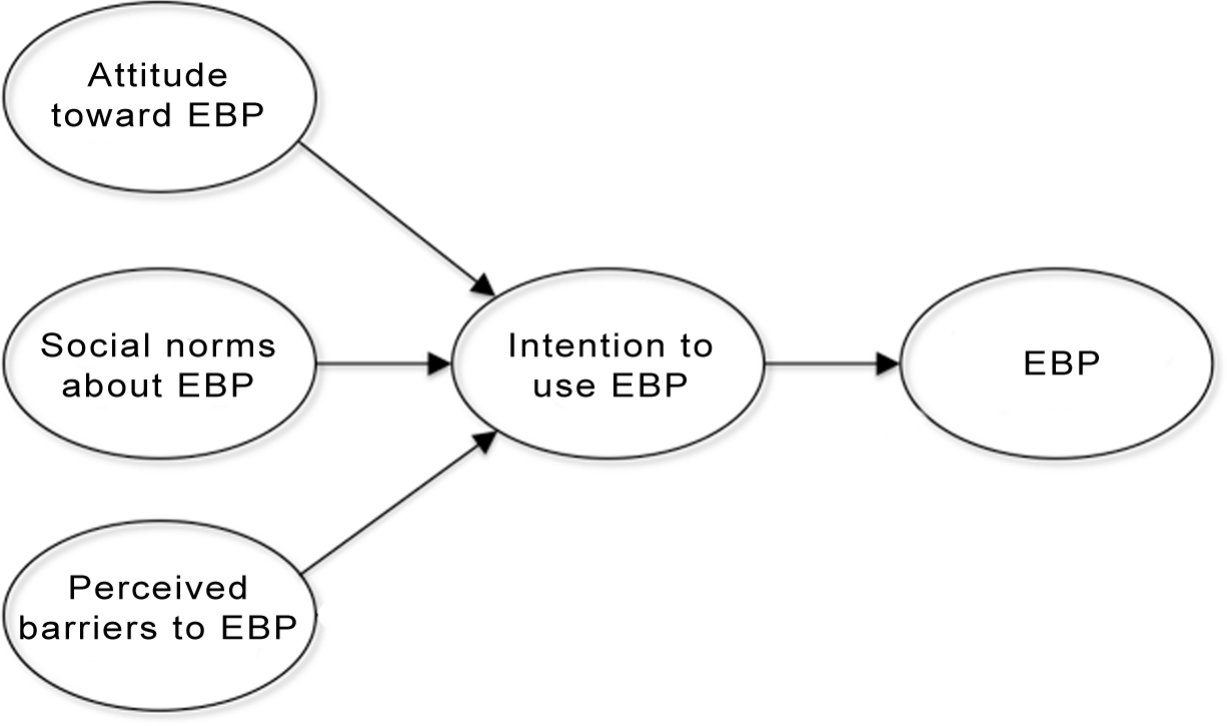
Theory of planned behavior (Ajzen).

Research identifies several reasons why practitioners across many areas of practice do not consult scientific evidence. Unfavorable individual attitudes and social norms espoused by peers often discourage practitioners from adopting practices based on scientific evidence [14–17]. Practitioner constraints also tend to limit use of EBP because of perceived barriers in their work settings hindering their ability to act in this way [18, 19]. These findings are consistent with the Theory of Planned Behavior [20] in which intended future behavior is a function of an individual’s attitudes toward that behavior (e.g., perceived benefits), the social norms surrounding it, and the personal (e.g., skills) and contextual factors (e.g., resources and time constraints) seen to facilitate or impede that behavior [21] (Fig 1)

Hence, we expect that practitioners are more likely to incorporate research evidence into their practice when they form positive attitudes towards scientific research, are exposed to supportive social norms regarding its use, and see the barriers to its use as surmountable. To facilitate the uptake of evidence-based practice (EBP), we thus need to know about practitioners’ attitudes, perceived social norms and barriers related to EBP. In management, such studies are scarce. In medicine and nursing, however, there is an abundance of research available on this subject, including several meta-analyses and systematic reviews comprehensively summarizing its findings [22–25]. As a result, we have opportunity to learn from these disciplines. If managers perceive similar barriers as physicians and nurses, this could reveal the kinds of strategies used successfully elsewhere to enhance the uptake of EBP in management.

A potential difficulty when comparing the attitudes of managers with those of practitioners in professions like medicine and nursing is the ambiguity regarding the term ‘manager’. The present study defines managers as individuals responsible for managing people and work in organizations. (REF Drucker). Nonetheless, the term ‘manager’ is difficult to operationalize objectively as those individuals managing people and work in organizations hold different titles and job descriptions. In addition, management in organizations is not a true profession. That is, the practice of management in organizations is not regulated, has no agreed-upon code of conduct or required knowledge base, and managers are not required to join a professional association. Objective inclusion criteria are hence not available. One means, however, through which practitioner attitudes towards EBP are shaped is by formal education. Although many managers lack formal management training, in the past decades the proportion of business and management students at universities has grown, with 25% of all master’s degrees in the U.S. being in business and management [26]. Thus, the present study uses the practitioner’s managerial education and/or membership in professional management associations as a basis for defining its sampling frame, rather than work domain (e.g. strategy, change management or human resources), organizational level (e.g. senior or mid-level management), or industry (for profit, public/private, sector).

## Research questions

To allow us to draw comparisons between managerial attitudes and perceived barriers to EBP and those of physicians and nurses, our research questions mirror those raised in the fields of medicine and nursing.

RQ 1: What evidence sources do managers report consulting in their daily practice?
RQ 2: What are managers’ attitudes towards the relevance and applicability of scientific research findings?
RQ 3: What are managers’ attitudes towards EBP?
RQ 4: What personal and contextual barriers do managers perceive to the use of scientific research findings?
RQ 5: Are managers’ attitudes towards EBP related to such background factors as
  ■ age?
  ■ education?
  ■ experience?
  ■ attention given to scientific research in their formal education?
  ■ experience in conducting scientific research?

## Method

### Questionnaire development

To draw comparisons with the field of medicine and nursing, we adapted and combined the widely used Barriers to Research Utilization Scale [27] and the McColl Questionnaire [28], since both measures are used in both domains and have sound psychometric properties for assessing attitudes and barriers related to EBP [29]. Additional questions were adapted from Rynes et al. [5]. The final survey consisted of 36 closed-ended questions along with several open-ended follow-up questions to assess the following topics: what evidence sources managers use in their daily routine, their familiarity with academic journals and online research databases, their experience in conducting research, the attention given to scientific research in their formal education, and personal demographic factors such as education and work experience. The questions used in the present study are available in the electronic supplement accompanying this article.

### Pre-testing

After we developed the initial questionnaire, we conducted a pilot study with a convenience sample of 74 Dutch interim managers in order to examine how managers interpreted our questions. We then reworded several items for clarity. For the convenience of the Belgian and Dutch managers, an independent native English speaker translated the questions into Dutch. The Dutch version was then back translated into English and checked against the original [30]. Our study was reviewed and approved by the Claremont Graduate University Institutional Review Board (application nr #1753).

### Sample and procedure

The British, Belgian, Dutch and Australian sampling frames were comprised of university alumni and members of professional organizations. The alumni were identified with the support of alumni officers from four Dutch universities and one Belgian and one Australian university. The professional members were identified through the secretarial office of the British and Belgian association for HR managers, and the Dutch association for interim-managers. The American sampling frame consisted of managers from the Leadership Library, a listing published by Leadership Directories, Inc. An email with an invitation to participate and a secure link to our online questionnaire was sent to a random sample of 30,000 U.S. managers. A similar email was sent to a convenience sample of 2,972 Dutch/Belgian managers, 2,785 Australian HR managers, and an active panel of 2,809 British HR managers. We emailed a reminder twice to all non-responders. The response rate for the American sample was 3% (n = 924), for the Belgian–Dutch sample 30% (n = 875), for the British sample 48% (n = 1,358), and for the Australian sample 7.5% (n = 210), giving an overall sample size of 3,367. One explanation for the low response rate of the American and Australian samples is incorrect email addresses since many emails were returned due to turnover or mistakes in the listing, making the true response rate likely much higher.

No sampling restrictions were applied regarding function (e.g. HR or finance), organizational level (e.g. senior or mid-level management), or industry. Two managerial groups–managers holding a second role a college or university teacher and managers with a previous career in academia– were excluded, since their research-related views and practices were less likely to be representative. This resulted in a final sample size of 2,789.

### Social desirability bias

Both the American and Dutch/Belgian survey tested the respondents’ knowledge of ten common research terms. One was a meaningless dummy term, which enabled us to test whether social desirability might bias responses. We examined the correlation and beta weight of this variable in relation to our criteria, and observed no effect.

### Missing data

We examined the nature of our missing data to determine if any further action was needed. Given that it was an on-line questionnaire, missing data occurred toward the latter part of questionnaire when respondents stopping filling it out. In the Australian and British sample, the overall percentage of missing data was limited (4% and 1%). In the Dutch, Belgian and American sample the rate of missing values per question ranged from 0% to 19.3% (mean 9.2%), thus emphasizing the need to choose an appropriate method for approximating missing values. We opted for the multiple imputation (MI) approach using the Markov chain Monte Carlo method, which yields less biased population estimates compared to traditional missing data methods [31].

## Results

Characteristics of the 2,789 respondents are described in Table 1. Because the British sample used a different scale for age these percentages are reported separately. These data were not gathered in the Australian sample.

**Table 1:** Demographics (%).

	Du/Be	US		UK	AUS
**Gender**					
male	58	63		45	36
female	41	37		55	64
**Age**					
< 30	14	1	< 25	1	na
30–39	28	6	25–34	13	na
40–49	28	20	35–44	27	na
50–59	25	45	45–54	31	na
> 60	6	29	> 55	28	na
**Education level**					
Bachelor's	17	23	Bachelor’s or Master’s	na	86
Master's	82	67			
Other	1	10	Other	na	14
**Experience**					
0–2	10	1		8	3
3–5	19	3		14	9
6–10	18	9		19	24
> 10	54	88		60	64

The large majority of the British and Australian respondents are employed in the domain of human resource management. The Dutch/Belgian and American managers on the other hand are employed in a wide range of work domains, including change, strategy and general management. An overview of the participants’ work domain is provided in Table 2.

**Table 2:** Work domain (%).

	Du/Be	US	UK	AUS
Human Resources	29	14	80	95
Marketing	11	18	20	na
Finance	17	18	32	na
Process	29	16	26	na
General	37	45	51	na
Strategy	39	29	na	na
Change	53	21	na	na
Quality	17	10	na	na
Customer services	na	na	28	na
ICT	na	na	13	na
Purchasing	na	na	22	na
R&D	na	na	15	na
Sales	na	na	17	na
Other	23	34	6	5

### RQ1: What evidence sources do managers consult in their daily practice?

As Table 3 indicates, most respondents report basing their decisions on personal experience (91%), intuition (64%), knowledge acquired through formal education (62%), advice from colleagues (59%), insights provided by experts (56%) or management literature (34%). Only a minority indicated that they often base their decisions on findings from scientific research (27%), and an even smaller minority (14%) had ever read a peer-reviewed academic journal. In addition, we asked respondents if they were familiar with online research databases relevant to management, such as ABI/INFORM, Business Source Elite, Science Direct or PsycINFO (see the questionnaire in the S1 Appendix for the question details). Results indicate that most managers are unfamiliar with (and thus do not use) online research databases.

**Table 3:** Evidence sources (percentages and weighted mean).

**On what do you base the decisions you make as a manager or consultant?**	Du/Be	US	UK	AUS	$\bar{X}w$
Personal experience	93	94	86	Na	**91**
Intuition	73	60	59	Na	**64**
Knowledge acquired through formal education	74	67	49	Na	**62**
Advice from a colleague	65	63	52	Na	**59**
Insights from experts	58	69	45	Na	**56**
Management literature	40	42	23	na	**34**
**How frequently do you consult scientific research literature when making decisions?**	Du/Be	US	UK	AUS	$\bar{X}w$
Always / Often	20	35	27	Na	**27**
Seldom/ Sometimes	54	42	57	Na	**52**
Never	23	24	14	Na	**20**

### RQ 2: What are managers’ attitudes towards the relevance and applicability of research findings?

Table 4 summarizes managers’ attitudes towards the relevance and applicability of research findings. Results suggest that a sizable group of respondents believe scientific research is interesting to managers and consultants (49%) and that the topics investigated are relevant to practice (51%). A majority disagreed with the statement that every organization is unique and that research findings would not apply to individual organizations (63%).

**Table 4:** Attitudes towards research findings (percentages and weighted mean).

Statement	Strongly agree/ somewhat agree					Strongly disagree/ somewhat disagree				
	Du/Be	US	UK	AUS	$\bar{X}w$	Du/Be	US	UK	AUS	$\bar{X}w$
Managers and consultants have no interest in scientific research.	33	14	33	10	**26**	45	67	37	64	**49**
Researchers investigate topics that are of no practical relevance.	21	21	26	9	**22**	54	57	41	61	**51**
Every organization is unique, hence the findings from scientific research are not applicable.	13	18	26	9	**18**	79	71	44	66	**63**

### RQ 3: What are managers’ attitudes towards EBP?

To assess attitudes regarding the general concept of EBP in the field of management, we provided respondents with a definition of EBP:

The conscientious, explicit, and judicious use of the best available evidence in making decisions about the management of individual organizations. The practice of evidence-based management means the integration of research evidence with individual managerial expertise in the context of organization characteristics, culture, and preferences.

As Table 5 indicates, most respondents (70%) claimed familiarity with the term. However, when the Dutch/Belgian and American respondents indicating familiarity with the term were asked to describe EBP in management, most did not answer or provided a limited answer (e.g. ‘Management based on research’). Using the definition provided, most respondents (69%) had positive attitudes towards EBP, and only a small minority (4%) had a negative attitude. Interestingly, most respondents perceived their colleagues’ attitudes towards EBP to be less favorable than their own. As Table 6 indicates, a large majority (73%) feel that managers can improve the quality of their work and advice to clients by using EBP. In addition, 62% agreed that in formal education more attention should be paid to EBP.

**Table 5:** Attitudes towards EBP (percentages and weighted mean).

Statement	Very positive/ positive					Very negative/ negative				
	Du/Be	US	UK	AUS	$\bar{X}w$	Du/Be	US	UK	AUS	$\bar{X}w$
How would you describe your attitude towards evidence-based practice?	67	68	71	71	**69**	6	4	3	2	**4**
How would you describe the attitude of most of your colleagues towards evidence-based practice?	24	39	54	43	**40**	25	13	6	15	**14**

**Table 6:** Attitudes towards EBP (percentages and weighted mean).

Statement	Strongly agree/ somewhat agree					Strongly disagree/ somewhat disagree				
	Du/Be	US	UK	AUS	$\bar{X}w$	Du/Be	US	UK	AUS	$\bar{X}w$
Evidence-based practice is not applicable to managers and consultants because their professions are based on hands-on experience and implicit knowledge.	15	11	16	12	**14**	65	70	56	59	**63**
Evidence-based practice does not do justice to the personal experience and implicit knowledge of managers and consultants.	14	17	23	17	**18**	61	53	38	46	**49**
By using evidence-based practices, managers can improve the quality of their work.	77	73	70	75	**73**	5	4	5	1	**4**
In the formal education of managers and consultants, more attention should be paid to evidence-based practice.	68	60	57	68	**62**	6	6	8	2	**6**

### RQ 4: What do managers perceive as personal or contextual barriers towards the use of research findings?

As Table 7 indicates, most respondents (58%) reported that they perceived lack of time to read research articles to be the main barrier to doing so. Examples of responses to the open questions include, “It is difficult to sit down, concentrate and find time to really read and digest.” This was followed by the perception that managers and consultants have little understanding of scientific research (51%) and that research articles are unreadable (37%). One explanation for the difference between the perceived unreadability between the Dutch/Belgian sample and the other samples could be the fact that they are non-native speakers of English, and thus a language barrier may be present. Other perceived barriers to using research findings mentioned in the open questions included organizational climate (“You need to be in a company that respects the need for research”), accessibility (“It is difficult to locate research papers, and I don’t know where to look”), and awareness (“I did not know research findings were available and accessible–are they?”).

**Table 7:** Perceived barriers (percentages and weighted mean).

Statement	Strongly agree/					Strongly disagree/				
	somewhat agree					somewhat disagree				
	Du/Be	US	UK	AUS	$\bar{X}w$	Du/Be	US	UK	AUS	$\bar{X}w$
Managers and consultants do not have enough time to read research articles	71	55	52	52	**58**	15	30	21	29	**22**
Managers and consultants have limited understanding of scientific research.	66	48	40	53	**51**	14	29	28	23	**24**
Research articles are unreadable.	55	32	30	15	**37**	27	43	41	57	**39**

### RQ 5: Are managers’ attitudes towards EBP related to their age, education, experience, attention given to science in their formal education, or their own experience in conducting research?

#### Age

A one-way ANCOVA was conducted to determine the degree of difference in attitudes across age groups controlling for professional experience and education. Only the American sample demonstrated a significant difference, though it was small, *F*(4, 548) = 2.610, *p* = .04, partial η2 = .02. Data on age were not available for the Australian sample. These findings suggest that attitudes towards EBP are not particularly associated with age.

#### Education

A one-way ANCOVA was conducted to determine the difference in attitudes across levels of education, controlling for professional experience. Both the Dutch/Belgium and American sample showed a significant difference, but this difference was very small, *F*(1, 649) = 5.02, *p* = .03, partial η2 = .008, and *F*(1, 552) = 5.06, *p* = .03, partial η2 = .009 respectively. The Australian sample showed a non-significant difference, *F*(1, 201) = 3.12, *p* = .08, partial η2 = .02. Data for the British sample were not available. These findings suggest that attitudes towards EBP are associated with education, but that its impact is small.

#### Experience

A one-way ANCOVA was conducted to determine the difference in attitudes across levels of experience, controlling for education. None of the four samples showed a significant difference (alpha values varying from .13 to .55) and all effect sizes were small (partial η2 varying from .005 to .03). These findings suggest that attitudes towards EBP are not associated with a manager’s professional experience.

#### Attention given to science

An independent t-test compared respondents whose formal education focused on scientific research with those whose education did not. The Dutch/Belgian, American and Australian sample all showed a significant difference at the .01 level, with the differences being small to medium (*d =* .31, 95% CI [.11–.50]; *d =* .*45*, 95% CI [.29–.61]; *d =* .*51*, 95% CI [.23–.79]. Data for the British sample were not available. This finding suggests that attitudes towards EBP are associated with the attention paid to science in the manager’s formal education, and that its impact is small to moderate.

#### Experience in conducting research

Finally, an independent t-test was conducted to compare respondents with and without experience in personally conducting research. The Dutch/Belgian, American and Australian sample all showed a significant difference at the .01 level, with small to medium effect sizes (*d* = .29, 95% CI [.13–.45]; *d* = .36, 95% CI [.13–.45]; *d* = .43, 95% CI [.16–.71]). Data for the British sample were not available. This finding suggests that attitudes towards EBP are associated with managers’ experience with conducting research, with its impact being small to moderate.

## Discussion

Our findings confirm conclusions of previous scholars regarding managerial attitudes towards evidence use, while also offering several new conclusions.

First, several researchers have suggested that when managers are looking for guidance on decisions, such as solving HR problems, they look first to the experience of their peers [5, 10, 32]. Our findings suggest that personal experience (94%), knowledge acquired through formal education (71%), and intuition (67%) tend to be their first source of evidence. This is consistent with the notion that managers fall back on the most readily available information [33].

Second, our study backs up research suggesting that managers are largely ignorant of findings from scientific research [5, 34–36]. Our findings indicates that only a minority of managers (33%) report that they base decisions on scientific research. This conclusion is also consistent with the results of systematic reviews in other disciplines, such as nursing [37] and education [23].

Third, our finding that most managers do not read academic journals (70%) aligns with the conclusions of previous research in various management domains [3, 5, 38, 39]. We also found that only a small proportion of managers (37%) are familiar with online research databases. As far as we are aware, there are no findings on this in similar studies in management, but a recent systematic review in healthcare suggests that among doctors and nurses this percentage is at least 88% [25]. Thus, there is an opportunity to raise managerial awareness and access to relevant research databases. Approaches to doing so include training business and professional management undergraduates and graduates in accessing databases and via alumni outreach by universities.

Scholars often assume that managers perceive scientific evidence as lacking in relevance (e.g.,[40–45]). However, our study challenges that assumption. Most respondents believe academic research to be relevant and perceive the topics researchers investigate to have practical value. These findings further reinforce the point that perceptions of scientific evidence may not inhibit practitioners nearly as much as access and other barriers.

The primary barriers to using research findings, indicated by our respondents, include perceived lack of time to read, the perception that managers have limited understanding of scientific research and the belief that research articles are unreadable are the main barriers to the use of research findings. These findings are consistent with the outcome of systematic reviews in medicine and nursing [24, 25, 37, 46–48]. In these reviews limited access to research evidence was perceived to be the third major barrier. However, the less frequent mention of this barrier by our respondents is remarkable given that research databases are only accessible for managers affiliated with educational institutions. That finding suggests that most managers may not even be aware of this limitation.

In addition, our finding that most respondents (69%) had positive attitudes towards EBP is comparable to the outcome of studies on practitioners’ attitudes in other disciplines. A recent systematic review based on 31 studies indicates that most healthcare professionals strongly believe EBP improves patient care and is important for their profession [25]. Other systematic reviews have similar findings, for example, 50 to 70% of physicians report positive attitude towards EBP [24, 47]. It is notable that our study finds a comparably positive attitude among managers despite the management’s lack of uptake in EBP.

Finally, we find that attitudes towards EBP are not associated with age or professional experience. However, these attitudes are somewhat affected by education and research experience. Experience with the research process has a small to moderate effect suggesting that it is the depth of research experience that matters in impacting attitude. For instance, organizations that conduct research themselves or participate in industry-university collaborations may afford their managers greater depth of experience with research and using its findings [49]. We also note that in medicine and nursing, demographic factors contributing to EBP attitudes are commonly studied, including age, education, work experience, and research experience. In these fields, systematic reviews have shown that effects on EBP attitudes from these demographic variables are evenly split as either positive or non-significant [18, 50–52].

## Implications for practice, education, and research

Our study suggests that most managers have positive attitudes towards EBP and that a large majority believes its use can improve the quality of their work. This positive perspective can provide leverage for educational institutions and advocates of EBP seeking to improve the managerial uptake of research findings and EBP. However, our study also indicates that most respondents perceive their *colleagues’* attitudes towards EBP to be less favorable than their own, which points to the possibility that, according to the Theory of Planned Behavior, social norms may limit the day-to-day practice of EBP [20]. An alternative explanation is that managers perceive themselves in a more socially desirable way than they do their colleagues, which is a well-known bias [53]. Such conditions suggest that it is likely to be easier for managers to engage in EBP where they are in senior positions within their organizations or where they work with like-minded others. Educating a new generation of managers to engage in EBP is important to the development of organizational cultures supporting evidence use.

Importantly, most managers we sampled appear to have an interest in research and believe that managerial research topics are of practical relevance. This is an important message to researchers, that practitioners may well be more receptive to their work than previously thought. The upshot is that it may be worthwhile to put serious effort into effectively communicating findings to practitioners, rather than solely focusing on other academics. We note, however, that since most managers do not read academic journals or access research databases, it is possible respondents are not sufficiently familiar with academic research to adequately answer questions about its relevance or value to their practice. Managers may have positive attitudes to research in general, but it is important to know how managers respond to actual research findings, particularly those that challenge their own practice [54].

Our study indicates that lack of time is perceived as the greatest barrier to the uptake of EBP in the field of management. It implies a role for senior management to promote decision-making practices that consider relevant scientific research along with other sources of evidence. This implication fits with a recent systematic review suggesting that supportive leadership and organizational climate are key factors in the implementation of EBP [55]. This finding also aligns with the Theory of Planned Behavior’s prediction that the implementation of EBP is partly determined by contextual supports and barriers. However, lack of time may also be a factor at the individual level, suggesting that managers have insufficient skills to swiftly read, appraise, and apply findings from research. This inference is supported by our finding that difficulty understanding and making sense of scientific research is perceived as the second biggest barrier to managers’ uptake of EBP. Educational institutions can play a major role in helping managers overcome this barrier by teaching management students how to read, critically appraise, and interpret research findings as part of the decision-making process.

Lack of time and understanding are barriers to address not only at end-user level (i.e. practitioners), but at the supplier level (i.e. scholars) as well. From the start of the EBP movement in the 1990s, it was clear that practitioners had little time or the skillset to regularly search for and appraise scientific evidence. For this reason, in disciplines where EBP is well established, pre-appraised evidence in the form of systematic reviews, rapid evidence assessments, or other types of evidence summaries written in plain English are provided by global communities such as the Cochrane and Campbell Collaborative, and by organizations such as the EPPI Centre. Such summaries enable practitioners to quickly consult the best available scientific evidence on issues of concern, check the research literature for new findings, and update their professional knowledge as new demands arise. Unfortunately in management, high-quality evidence summaries not yet widely available, and as a result, neither the management academic nor the management practitioner can claim to be well-informed regarding research findings [56]. Even today many management academics remain uncertain as to the value of evidence summaries,or unconvinced about their practical or academic value [57]. This unfortunate situation results partly because the education of future academics focuses solely on techniques required to conduct primary research and to present this work to other academics [58]. This narrow focus ignores the essential skills needed to critically appraise and summarize the best available evidence on a topic relevant for practice, and to communicate this information in ways that are comprehensible to lay audiences. Hence, when it comes to breaking down barriers impeding EBP in the field of management, universities and PhD programs, in particular, play an important role.

## Limitations

Despite achieving some notable insights, the present study has its limitations. Our sample was not random, but based on populations in which individuals identified themselves as managers or leaders. In addition, the response rate of our survey for the American sample was just 3%, and the response rates for the Belgian-Dutch, British and Australian sample were 30%, 48% and 7.5% respectively. We do not have information about the managers and consultants who did not respond. Their lack of response may have been due to a negative attitude towards the use of research findings or skepticism towards EBP. All of this makes our findings prone to selection bias.

Another limitation of our survey concerns the definition of both ‘management literature’ and ‘scientific literature’. In general, scientific literature refers to peer-reviewed research published in academic journal, textbooks or white papers. Management literature, on the other hand, includes commercial business books or magazines that typically don’t make use of findings from empirical studies. It is unclear, however, whether all respondents were familiar with this distinction.

An important shortcoming of our survey is the definition of the target population. As mentioned above, various definitions of the term ‘manager’ are used in the literature. Practitioners in management hold diverse titles and job descriptions. The common denominator for our participants is their level of education. It is true that great differences exist between bachelor and master programs in management [59], however, such differences are present in the education of physicians and nurses too, and as a result medical practice is far from uniform [60–62]. Given our participants’ background, our findings may not be representative of all holding the title of manager or performing managerial work, however, they are likely to be representative of those managers with formal management education.

## Conclusions

Our findings indicate that most managers we sampled have positive attitudes towards EBP. However, lack of time and a limited understanding of scientific research are seen as major barriers to managers’ uptake and implementation of EBP. Notably, studies within other professions where EBP is far more established report the same barriers. These findings suggest that managers tend to follow a pattern common among practitioners in other fields.

To enhance the use and adoption of EBP by managers, organizational leaders need to promote a climate that promotes awareness of scientific research and use of scientific evidence as part of the decision-making process. Educational institutions should focus on improving the EBP skills needed to understand, search for and apply scientific evidence. Finally, greater effort on the part of educators and scholars is needed to summarize relevant management research in practitioner-friendly form. Only when these three conditions are met, will managers be able to overcome the key barriers impeding uptake of EBP in the field of management.

## Supporting information

S1 Appendix Survey Questions (English). (PDF)

S2 Appendix Survey Questions (Dutch). (PDF)
