# Supplementary material for: Managerial attitudes and perceived barriers regarding evidence-based practice: An international survey
Source: PLoS One. 2017 Oct 3;12(10):e0184594. doi: 10.1371/journal.pone.0184594 (PMC5626028; doi:10.1371/journal.pone.0184594)
Supplement: S1 Appendix — (PDF) [file pone.0184594.s001.pdf]

Survey Questions Attitudes and Barriers Towards Evidence-Based Management

First we would like to ask you a few questions about your job and yourself.

What is your gender?

☐ male ☐ female

What is your age?

☐ < 30 years
☐ 30 - 39 years
☐ 40 - 49 years
☐ 50 - 59 years
☐ ≥ 60 years

How many years of experience do you have in the field of management and/or consulting?

☐ student
☐ 0 to 2 years
☐ 3 to 5 years
☐ 6 to 10 years
☐ over 10 years

What is your current occupation group?

☐ External consultant
☐ Internal consultant
☐ Manager
☐ Other

What is your highest level of education?

☐ Doctoral Degree
☐ Master Degree
☐ Bachelor's degree
☐ Professional degree
☐ Other

In which area of expertise are you employed? (more than one area is possible)

- ☐ Strategy
- ☐ Management of change
- ☐ Process improvement
- ☐ Human resources
- ☐ Other
- ☐ Finance
- ☐ Marketing
- ☐ Quality management
- ☐ General management

Part 1

In this part, we would like to ask you some questions about your daily routine as a manager or consultant.

1. On what do you base the decisions you make as a manager or consultant?

(more than one answer is possible)

- ☐ intuition
- ☐ personal experience
- ☐ knowledge acquired through formal education
- ☐ insights provided by experts
- ☐ advice from a colleague
- ☐ management literature
- ☐ results from scientific research
- ☐ other

2. How often do you consult each source of knowledge in your daily routine as a manager or consultant?

	Never	Seldom	Often	Daily
colleagues within your organization	☐	☐	☐	☐
colleagues outside of your organization	☐	☐	☐	☐
external consultants	☐	☐	☐	☐
(former) teachers or professors	☐	☐	☐	☐
internet	☐	☐	☐	☐
management literature	☐	☐	☐	☐
scientific research literature	☐	☐	☐	☐

3. Are there knowledge sources which you use which have not been mentioned above?

☐ No

☐ Yes, ……

Part 2

In this part we would like to ask you questions regarding your use of professional literature.

4. With which of the following management literature are you familiar?

	Unknown	Known, but never read	Known, occasionally read	Known, frequently read
Bloomberg BusinessWeek	☐	☐	☐	☐
Forbes	☐	☐	☐	☐
Fast Company	☐	☐	☐	☐
Inc.	☐	☐	☐	☐
Strategy & Business	☐	☐	☐	☐
Academy of Management Review	☐	☐	☐	☐
Academy of Management Journal	☐	☐	☐	☐
Harvard Business Review	☐	☐	☐	☐
Strategic Management Journal	☐	☐	☐	☐
Strategy & Business	☐	☐	☐	☐
Journal of Management	☐	☐	☐	☐
Administrative Science Quarterly	☐	☐	☐	☐
MIT Sloan Management Review	☐	☐	☐	☐
Organization Science	☐	☐	☐	☐
Leadership Quarterly	☐	☐	☐	☐

5. Are there management journals which you read which have not been listed?

☐ No

☐ Yes, ……

Part 3

In this part we would like to ask you questions regarding your use of research articles. Research articles are articles within a professional journal in which the results of scientific research are described.

6. How many times have you searched for research articles on the internet or in a library over the past 6 months? (please provide best estimate)

   ☐ times

7. With which of the online databases are you familiar? (more than one answer is possible)

☐ ABI/INFORM from ProQuest
☐ Business Source Premier from EBSCO
☐ Science Direct from Elsevier
☐ PsycINFO
☐ ISI Web of Knowledge
☐ Google Scholar
☐ other
☐ I'm not familiar with online databases (***proceed to question 10***)

8. How many times have you searched for research articles in these online databases in the past 6 months? (please provide best estimate)

   ☐ times

9. How many times have you read a research article in the past 6 months which influenced your daily routine as manager or consultant? (please provide best estimate)

   ☐ times

- What was the topic(s)? ………… (list as many topics as you can remember)

Part 4

In this part, we would like to ask you questions regarding the application of scientific research.

10. Do you have experience conducting scientific research?

☐ Yes

☐ No

11. Was there special attention given to scientific research in your formal education?

☐ Yes

☐ No

12. How familiar are you with the following research terms?

	Unknown	Somewhat known	Known, and I could explain it to others
controlled study	☐	☐	☐
observational study	☐	☐	☐
case study	☐	☐	☐
confidence interval	☐	☐	☐
statistical significance	☐	☐	☐
internal validity	☐	☐	☐
reliability	☐	☐	☐
densitivity	☐	☐	☐
generalizability	☐	☐	☐
bias	☐	☐	☐
correlation	☐	☐	☐

13. What percentage of your daily routine as a manager or consultant do you feel is based on findings from scientific research?

….. %

Part 5

In this part of the survey a number of statements are put forward which explain why scientific research might not be applied in practice. You are asked to give your opinion on the truthfulness of these statements.

14. Every organization is unique, hence the findings from scientific research are not applicable.

- ☐ Strongly agree
- ☐ Somewhat agree
- ☐ Neither agree or disagree
- ☐ Somewhat disagree
- ☐ Strongly disagree

15. The results of scientific research are theoretically sound, but do not work in practice.

- ☐ Strongly agree
- ☐ Somewhat agree
- ☐ Neither agree or disagree
- ☐ Somewhat disagree
- ☐ Strongly disagree

16. Scientific research is conducted by researchers who are too far removed from the day-to-day work of a practitioner.

- ☐ Strongly agree
- ☐ Somewhat agree
- ☐ Neither agree or disagree
- ☐ Somewhat disagree
- ☐ Strongly disagree

17. Researchers investigate topics which have no practical relevance.

- ☐ Strongly agree
- ☐ Somewhat agree
- ☐ Neither agree or disagree
- ☐ Somewhat disagree
- ☐ Strongly disagree

18. Managers and consultants do not have enough time to read research articles.

☐ Strongly agree
☐ Somewhat agree
☐ Neither agree or disagree
☐ Somewhat disagree
☐ Strongly disagree

19. Managers and consultants have limited understanding of scientific research.

☐ Strongly agree
☐ Somewhat agree
☐ Neither agree or disagree
☐ Somewhat disagree
☐ Strongly disagree

20. Managers and consultants are practitioners and therefore have no interest in scientific research.

☐ Strongly agree
☐ Somewhat agree
☐ Neither agree or disagree
☐ Somewhat disagree
☐ Strongly disagree

21. Research articles are unreadable.

☐ Strongly agree
☐ Somewhat agree
☐ Neither agree or disagree
☐ Somewhat disagree
☐ Strongly disagree

22. Are there any other reasons you can think of to explain why scientific research might not be used by managers and consultants?

☐ No
☐ Yes

Part 6

In this part we would like to ask you some questions regarding evidence-based management.

23. Are you familiar with the term evidence-based management?

☐ Yes
☐ Somewhat
☐ No **(skip to text)**

- How would you describe evidence-based management?

text:

Evidence-based management is often described as "*the conscientious, explicit, and judicious use of the best available evidence in making decisions about the management of individual organizations. The practice of evidence based management means the integration of research evidence with individual managerial expertise in the context of organization characteristics, culture and preferences.*"

Next, a couple of questions and statements regarding your current attitudes towards evidence-based management are put forward. You are asked to give your opinion on the truthfulness of these statements.

24. How would you describe your attitude towards evidence-based management?

very positive ☐ ☐ ☐ ☐ ☐ very negative

25. How would you describe the attitude of most of your colleagues towards evidence-based management?

very positive ☐ ☐ ☐ ☐ ☐ very negative

26. Evidence-based management is not applicable for managers and consultants because their professions are based on hands-on experience and implicit knowledge.

strongly agree ☐ ☐ ☐ ☐ ☐ strongly disagree

27. Evidence-based management does not do justice to the personal experience and implicit knowledge of managers and consultants.

strongly agree ☐ ☐ ☐ ☐ ☐ strongly disagree

28. By using evidence-based practices, consultants can improve the quality of their advice to clients.

strongly agree ☐ ☐ ☐ ☐ ☐ strongly disagree

29. By using evidence-based practices, managers can improve the quality of their work.

strongly agree ☐ ☐ ☐ ☐ ☐ strongly disagree

30. In the formal education of managers and consultants, more attention should be paid to evidence-based management.

strongly agree ☐ ☐ ☐ ☐ ☐ strongly disagree

Thank you very much for your cooperation!

If you are interested in the results of the investigation, you can fill in your email address below.
