# Supplementary material for: Managerial attitudes and perceived barriers regarding evidence-based practice: An international survey
Source: PLoS One. 2017 Oct 3;12(10):e0184594. doi: 10.1371/journal.pone.0184594 (PMC5626028; doi:10.1371/journal.pone.0184594)
Supplement: S2 Appendix — (PDF) [file pone.0184594.s002.pdf]

Deel 1

Eerst willen we u een aantal vragen stellen over uw dagelijks werk als manager of consultant

1. Op basis waarvan neemt u beslissingen tijdens uw dagelijks werk als manager of consultant?

(meerdere antwoorden mogelijk)

- ☐ intuïtie
- ☐ eigen ervaring
- ☐ kennis opgedaan tijdens een opleiding of cursus
- ☐ inzichten van experts of deskundigen
- ☐ advies van collega's
- ☐ management literatuur
- ☐ uitkomst van wetenschappelijk onderzoek
- ☐ anders, namelijk …..

2. Van welke kennisbronnen maakt u gebruik tijdens uw dagelijks werk als manager of consultant?

	Nooit	Zelden	Vaak	Dagelijks
Collega's binnen uw organisatie	☐	☐	☐	☐
Collega's buiten uw organisatie	☐	☐	☐	☐
Externe consultants	☐	☐	☐	☐
(Voormalige) leraren of docenten	☐	☐	☐	☐
Internet	☐	☐	☐	☐
Management literatuur	☐	☐	☐	☐
Wetenschappelijke onderzoeksliteratuur	☐	☐	☐	☐

3. Zijn er kennisbronnen die u gebruikt die hierboven niet genoemd worden?

- ☐ Nee
- ☐ Ja, namelijk:

Deel 2

In dit deel willen we u een aantal vragen stellen over het gebruik van vakliteratuur

4. Welke van de onderstaande managementtijdschriften kent u?

	Onbekend	Bekend, nog nooit gelezen	Bekend, wel eens gelezen	Bekend, vaak gelezen
Management Team	☐	☐	☐	☐
FEM Business	☐	☐	☐	☐
Holland Management Review (HMR)	☐	☐	☐	☐
Management en Organisatie (M&O)	☐	☐	☐	☐
Maandblad voor Accounting en Bedrijfskunde (MAB)	☐	☐	☐	☐
Academy of Management Review	☐	☐	☐	☐
Academy of Management Journal	☐	☐	☐	☐
Harvard Business Review	☐	☐	☐	☐
Strategic Management Journal	☐	☐	☐	☐
Strategy & Business	☐	☐	☐	☐
Journal of Management	☐	☐	☐	☐
Administrative Science Quarterly	☐	☐	☐	☐
MIT Sloan Management Review	☐	☐	☐	☐
Organization Science	☐	☐	☐	☐
Leadership Quarterly	☐	☐	☐	☐

5. Zijn er managementtijdschriften die u leest die hierboven niet genoemd worden?

☐ Nee

☐ Ja, namelijk:

Deel 3

In dit deel willen wij u een aantal vragen stellen over het gebruik van onderzoeksartikelen. Onderzoeksartikelen zijn publicaties in vakliteratuur waarin de uitkomst van wetenschappelijk onderzoek wordt beschreven.

6. Hoe vaak heeft u in het afgelopen jaar op internet of in een bibliotheek gezocht naar onderzoeksartikelen?

☐ 0 keer

☐ 1 tot 2 keer

☐ 3 tot 4 keer

☐ meer dan 4 keer

7. Met welke online database bent u bekend? (meerdere keuzes mogelijk)

☐ ABI/INFORM van ProQuest

☐ Business Source Premier van EBSCO

☐ Science Direct van Elsevier

☐ PsycINFO

☐ ISI Web of Knowledge

☐ Google Scholar

☐ anders, namelijk …..

☐ ik ben niet bekend met online databases (***ga door naar vraag 10***)

8. Hoe vaak heeft u in het afgelopen jaar in deze database(s) gezocht naar onderzoeksartikelen?

☐ 0 keer

☐ 1 tot 2 keer

☐ 3 tot 4 keer

☐ meer dan 4 keer

9. Hoe vaak heeft u in het afgelopen jaar een onderzoeksartikel gelezen waarvan de uitkomst van invloed is geweest op uw dagelijks werk als manager of consultant?

☐ 0 keer

☐ 1 tot 2 keer*

☐ 3 tot 4 keer*

☐ meer dan 4 keer*

* Wat was het onderwerp?

Deel 4

In dit deel willen wij u een aantal vragen stellen over de toepassing van wetenschappelijk onderzoek.

10. Heeft u ervaring met het doen van wetenschappelijk onderzoek?

☐ Ja

☐ Nee

11. Is in de opleiding die u gevolgd heeft aandacht besteed aan wetenschappelijk onderzoek?

☐ Ja

☐ Nee

12. Welke van de onderstaande onderzoekstermen kent u?

	Onbekend	Enigszins bekend	Bekend, en ik kan het uitleggen aan anderen
gecontroleerd onderzoek	☐	☐	☐
observationeel onderzoek	☐	☐	☐
kwalitatief onderzoek	☐	☐	☐
betrouwbaarheidsinterval	☐	☐	☐
significantie	☐	☐	☐
interne validiteit	☐	☐	☐
betrouwbaarheid	☐	☐	☐
densiviteit	☐	☐	☐
representativiteit	☐	☐	☐
bias	☐	☐	☐
correlatie	☐	☐	☐

13. Welk percentage van uw dagelijkse praktijk als manager of consultant is volgens u gebaseerd op inzichten die zijn voortgekomen uit wetenschappelijk onderzoek?

….. %

Deel 5

Nu volgen een aantal stellingen over belemmeringen voor de toepassing van inzichten gebaseerd op wetenschappelijk onderzoek in de praktijk

14. De kloof tussen wetenschap en de dagelijkse praktijk van managers en consultants is groot

- ☐ Helemaal mee eens
- ☐ Enigszins mee eens
- ☐ Noch mee eens, noch mee oneens
- ☐ Enigszins mee oneens
- ☐ Helemaal mee oneens

15. Iedere organisatie is uniek, daarom is de uitkomst van wetenschappelijk onderzoek niet toepasbaar

- ☐ Helemaal mee eens
- ☐ Enigszins mee eens
- ☐ Noch mee eens, noch mee oneens
- ☐ Enigszins mee oneens
- ☐ Helemaal mee oneens

16. Uitkomst van wetenschappelijk is theoretisch juist, maar werkt niet in de praktijk

- ☐ Helemaal mee eens
- ☐ Enigszins mee eens
- ☐ Noch mee eens, noch mee oneens
- ☐ Enigszins mee oneens
- ☐ Helemaal mee oneens

17. Wetenschappelijk onderzoek wordt gedaan door onderzoekers die te ver van de praktijk af staan

- ☐ Helemaal mee eens
- ☐ Enigszins mee eens
- ☐ Noch mee eens, noch mee oneens
- ☐ Enigszins mee oneens
- ☐ Helemaal mee oneens

18. Onderzoekers doen wetenschappelijk onderzoek naar onderwerpen die niet relevant zijn voor de praktijk

- ☐ Helemaal mee eens
- ☐ Enigszins mee eens
- ☐ Noch mee eens, noch mee oneens
- ☐ Enigszins mee oneens
- ☐ Helemaal mee oneens

19. Managers en consultants hebben te weinig tijd om onderzoeksartikelen te lezen

- ☐ Helemaal mee eens
- ☐ Enigszins mee eens
- ☐ Noch mee eens, noch mee oneens
- ☐ Enigszins mee oneens
- ☐ Helemaal mee oneens

20. Managers en consultants hebben te weinig kennis van wetenschappelijk onderzoek

- ☐ Helemaal mee eens
- ☐ Enigszins mee eens
- ☐ Noch mee eens, noch mee oneens
- ☐ Enigszins mee oneens
- ☐ Helemaal mee oneens

21. Managers en consultants zijn praktijkmensen en daarom niet geïnteresseerd in wetenschappelijk onderzoek

- ☐ Helemaal mee eens
- ☐ Enigszins mee eens
- ☐ Noch mee eens, noch mee oneens
- ☐ Enigszins mee oneens
- ☐ Helemaal mee oneens

22. Onderzoeksartikelen zijn moeilijk leesbaar

- ☐ Helemaal mee eens
- ☐ Enigszins mee eens
- ☐ Noch mee eens, noch mee oneens
- ☐ Enigszins mee oneens
- ☐ Helemaal mee oneens

23. Zijn er volgens u nog andere belemmeringen voor de toepassing van inzichten gebaseerd op wetenschappelijk onderzoek in de dagelijkse praktijk van managers en consultants?

- ☐ Nee
- ☐ Ja, namelijk ……

Deel 6

In dit deel willen wij u een aantal vragen stellen over Evidence Based Management

24. Bent u bekend met het begrip Evidence Based Management?

- ☐ Ja*
- ☐ Een beetje*
- ☐ Nee

* Hoe zou u Evidence Based Management omschrijven?

Evidence-based management wordt vaak omschreven als: *“het expliciet en oordeelkundig gebruik van het beste wetenschappelijke bewijsmateriaal om management-beslissingen te nemen voor individuele organisaties. Bij evidence-based management wordt de uitkomst van wetenschappelijk onderzoek geïntegreerd met de individuele kennis en ervaring van de manager / consultant en de specifieke context, voorkeuren en karakteristieken van de organisatie.”*

Hieronder volgen een aantal vragen en stellingen over uw houding ten opzichte van evidence based management.

25. Hoe zou u uw houding ten opzichte van evidence based management omschrijven?

Zeer positief ☐ ☐ ☐ ☐ ☐ zeer negatief

26. Hoe zou u de houding van het merendeel van uw collega’s ten opzichte van evidence based management omschrijven?

Zeer positief ☐ ☐ ☐ ☐ ☐ zeer negatief

27. Evidence based management is niet bruikbaar voor managers en consultants omdat hun vak voornamelijk gebaseerd is op ervaring en impliciete kennis.

Geheel mee eens ☐ ☐ ☐ ☐ ☐ Geheel mee oneens

28. Evidence based management doet geen recht aan de persoonlijke ervaring en impliciete kennis managers en consultants

Geheel mee eens ☐ ☐ ☐ ☐ ☐ Geheel mee oneens

29. Door evidence based te werken kan een consultant de kwaliteit van zijn adviezen verbeteren

Geheel mee eens ☐ ☐ ☐ ☐ ☐ Geheel mee oneens

30. Door evidence based te werken kan een manager de kwaliteit van zijn werk verbeteren

Geheel mee eens ☐ ☐ ☐ ☐ ☐ Geheel mee oneens

31. In de opleiding van managers en consultants zou meer aandacht besteed moeten worden aan evidence based management

Geheel mee eens ☐ ☐ ☐ ☐ ☐ Geheel mee oneens

Deel 7

Tot slot willen wij u een aantal vragen stellen over uw werk en uzelf

Wat is uw geslacht?

☐ man ☐ vrouw

Wat is uw leeftijd?

…. jaar

Hoeveel jaar ervaring heeft u op het gebied van management en/of consultancy?

☐ student
☐ 0 tot 2 jaar
☐ 3 tot 5 jaar
☐ 6 tot 10 jaar
☐ meer dan 10 jaar

Wat is uw huidige werksituatie?

☐ Zelfstandig adviseur
☐ Adviseur werkzaam bij een adviesbureau
☐ Intern adviseur werkzaam bij een bedrijf of organisatie
☐ Interim manager
☐ Manager bij een bedrijf of organisatie
☐ anders, namelijk:

Wat is uw hoogst genoten opleiding?

☐ HBO
☐ Universiteit
☐ Business School
☐ Post doctorale opleiding
☐ anders, namelijk:

Op welk deelgebied bent u werkzaam (meerdere keuzes mogelijk)

- ☐ Strategie
- ☐ Financiën
- ☐ Marketing
- ☐ Verandermanagement
- ☐ Proces optimalisatie
- ☐ Kwaliteitsmanagement
- ☐ HRM
- ☐ Algemeen management
- ☐ anders, namelijk:

Hartelijk dank voor uw medewerking!

Indien u op de hoogte gesteld wil worden van de uitkomst van het onderzoek en in aanmerking wil komen voor de ballonvaart kunt u hieronder uw naam, adres en e-mailadres invullen.
